# Gene Expression of Axon Growth Promoting Factors in the Deer Antler

**DOI:** 10.1371/journal.pone.0015706

**Published:** 2010-12-20

**Authors:** Wolfgang Pita-Thomas, Carmen Fernández-Martos, Mónica Yunta, Rodrigo M. Maza, Rosa Navarro-Ruiz, Marcos Javier Lopez-Rodríguez, David Reigada, Manuel Nieto-Sampedro, Manuel Nieto-Diaz

**Affiliations:** 1 Experimental Neurology Unit, Hospital Nacional de Parapléjicos (SESCAM), Toledo, Spain; 2 Neural Plasticity Lab, Instituto Cajal de Neurociencias (CSIC), Madrid, Spain; National Institute on Aging (NIA), National Institutes of Health (NIH), United States of America

## Abstract

The annual regeneration cycle of deer (Cervidae, Artiodactyla) antlers represents a unique model of epimorphic regeneration and rapid growth in adult mammals. Regenerating antlers are innervated by trigeminal sensory axons growing through the velvet, the modified form of skin that envelopes the antler, at elongation velocities that reach one centimetre per day in the common deer (*Cervus elaphus*). Several axon growth promoters like NT-3, NGF or IGF-1 have been described in the antler. To increase the knowledge on the axon growth environment, we have combined different gene-expression techniques to identify and characterize the expression of promoting molecules not previously described in the antler velvet. Cross-species microarray analyses of deer samples on human arrays allowed us to build up a list of 90 extracellular or membrane molecules involved in axon growth that were potentially being expressed in the antler. Fifteen of these genes were analysed using PCR and sequencing techniques to confirm their expression in the velvet and to compare it with the expression in other antler and skin samples. Expression of 8 axon growth promoters was confirmed in the velvet, 5 of them not previously described in the antler. In conclusion, our work shows that antler velvet provides growing axons with a variety of promoters of axon growth, sharing many of them with deer's normal and pedicle skin.

## Introduction

The capability to regenerate large sections of the body plan is typical of some invertebrates and urodele amphibians, while in mammals it is almost restricted to organs like the skin or the exceptional deer antlers [1], [2]. Every year, male deers shed (cast) their antlers and fulfill a complete regeneration process that leads to the formation of a new set of antlers in approximately three months. The growing antler is an extension of the antler pedicle periostium [3] that proliferates and differentiates into cartilage and bone tissue to form the bone core of the new antlers. Growing antlers are enveloped in a hair-covered skin known as velvet that presents several peculiarities, including lack of sweat glands and *arrector pili* muscles and the presence of abundant multilobullated sebaceous glands[4]. Antlers are innervated by sensory branches of the trigeminal nerve [5] that enter the antler in association to blood vessels, at the vascular layer of the velvet [6], [7], [8], [9]. At the end of the summer, antlers become calcified and velvet sheds, leaving the bony core used in agonistic encounters during the rut season.

Antlers are a valuable model to study mechanisms of organ regeneration and rapid tissue growth [10], [11]. However, antler innervation has received little attention, even though it can inform us on mechanisms underlying neuron survival after large and prolonged denervations or axonal regeneration in adult mammals. Antler innervation is also noted for its rapid growth [7], reaching elongation rates over two centimeters per day in the moose [12] or 1 cm/day in the red deer [13]. Little is known about the factors responsible for this rapid growth. Whole antler extracts promote neurite outgrowth *in vitro* [14] and we have shown that velvet -but not mesenchyme- secretes nerve growth factor (NGF) and other molecules that strongly promote neurite outgrowth *in vitro* [9]. On the other hand, velvet substrata (extracellular matrix and cell membranes) shows axonal guiding properties [9]. These observations suggest a paracrine regulation of the antler nerve growth. In agreement, several axon growth promoters have been described in the antler, including growth factors like neurotrophin 3 (NT-3) [15], NGF [8], Vascular endothelial growth factor (VEGF) and Pleiotrophin [16], insulin growth factor 1(IGF-I) [17], [18], transforming growth factor beta (TGFβ[18], Bone Morphogenetic Proteins (BMPs) [19], [20], [21], and extracellular matrix components like collagen, laminin or heparan sulfate [22], [23].

In the present study we have combined microarray, RT-PCR and sequencing analyses to identify axon growth promoters not previously described in the antler velvet. Microarray analysis allowed us to hypothesize the expression or changes in expression of 90 promoters and/or regulators of the axonal growth. 15 of them were sequenced and analyzed by quantitative Real Time RT-PCR (qPCR), establishing the expression in the antler velvet of B*rain derived neurotrophic factor (BDNF)*, G*lucose phosphate isomerase (GPI)*, M*eteorin (MTRN)*, *Midkine (MDK)*, or *Neuronal cell adhesion molecule(NRCAM)* previously not observed in deer, together with *Fibroblast growth factor (FGF2)*, *BMP2* and *TGFβ*. We compared the gene expression of these promoters in the velvet with the expression in the mesenchyme and in samples of unmodified skin covering the antler pedicle and the frontal bone. Expression profiles showed that several growth promoters were overexpressed in velvet with respect to mesenchyme but not to skin samples, *Midkine* being the most significant exception. Integration of these and previous data allowed us to draw a more complete picture of the growth promoting environment of the antler innervation during its annual regrowth.

## Results

### Gene expression profiles of deer antler tissues

**Figure 1:**
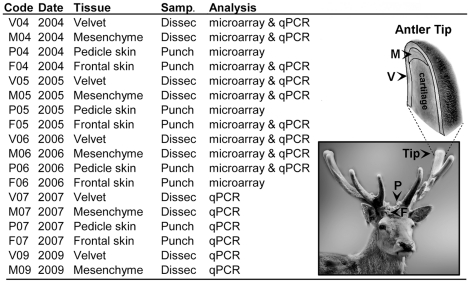
Samples employed in the study. For every sample, the table details its code, year of sampling, type of tissue sampled, sampling method, and the analyses in which it was used. The associated photographs show the sampling areas V: Velvet; M: Mesenchyme; P: Pedicle skin and F: Frontal skin. Punch samples were obtained using a biopsy punch while Dissec ones were obtained after dissection of the antler tissues. qPCR corresponds to quantitative real-time PCR analysis.

**Figure 2:**
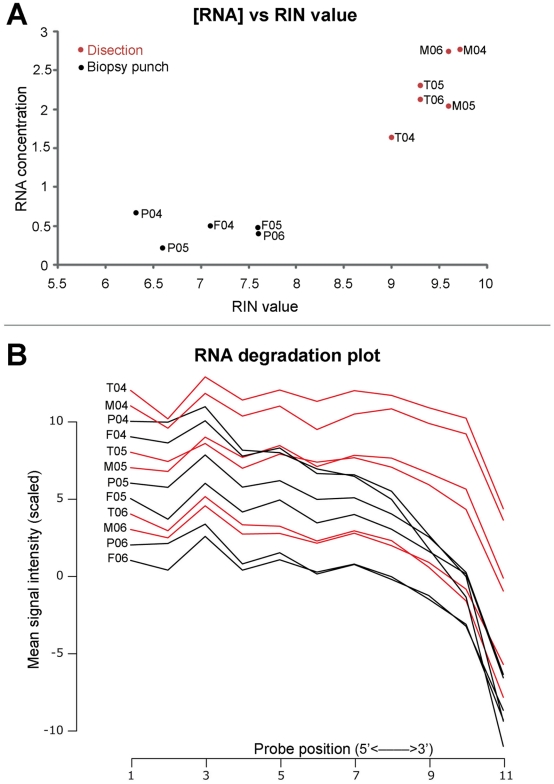
RNA quality measurements of the studied samples. Figure details different RNA quality measurements obtained before (A) and after microarray hybridization (B). A) Relationship between quality, measured as the RNA integrity number (RIN algorithm from Agilent), and concentration (mg/ml) of the total RNA before hybridization or PCR. Graph shows a clear separation between pedicle and frontal samples obtained using a biopsy punch and those obtained after dissection of the antler tissues. B) The degradation plot indicates the mean scales values of the different probes for all probesets in the Affymetrix arrays. As indicated in the x axis, probes are ordered according to their 5′ to 3′ position in the RNA sequence. RNA degradation tends to start at the 3′ extreme and to proceed to the 5′ extreme. The graph shows some level of degradation but does not reflect the previously observed separation between punch (black lines) and dissection obtained samples (red lines). Thus, it seems that the RNA quality differences observed prior to hybridization are not reflected by the hibridization values. Sample codes as in figure 1.

Only a few of the molecules claimed to regulate axonal growth have been studied in the deer antler. To screen for growth promoters in the antler velvet, we performed gene expression analyses using DNA microarrays. A very small number of cervid mRNA sequences have been described (296 deposited at the NCBI in January 2010 [24]), making species specific microarrays unavailable. Several authors have proposed that cross-species microarray analysis can provide valid and reproducible information given that the species being analyzed is enough closely related to the species used to construct the array. Since previous studies have proposed that the sequence divergence and protein structure similarity among mammals will guarantee valid results [25], we used the well known and highly annotated Affymetrix human U133plus 2 Genechip to carry out the gene expression profiling. Total RNA of velvet, mesenchyme, pedicle skin and frontal skin from 3 adult male red deers (*Cervus elaphus*) were hybridized to the microarrays (see Figure 1). RNA quantity and integrity varied among sample types. Velvet and mesenchyme produced higher RNA yields and better quality (see Figure 2) than pedicle and frontal skin, although, quality of all RNA samples was enough to perform the analyses according to Affymetrix standards. In fact, differences in RNA quality did not have any clear correspondence in all but one of the quality measures based on the hybridization data. RNA degradation plots seemed to reflect to some extent the bimodality between samples types although individual variability was clearly larger than the variations caused by the sample type (see Figure 2). In all samples, the percentage of present calls was below the 10% of the transcripts being analysed. These values are clearly below the ones obtained when hybridizing human samples (40-50%), in agreement with the known decrease in sensibility of the cross species analyses [26].

Raw prove-level data (.CEL Files) obtained after hybridizing the deer samples to the Affymetrix U133plus 2 GeneChip are available at the NCBI GEO database [27] under accession number GSE20036. Microarray data analysis was performed using different processing (background subtraction, normalization, summarizing) approaches in order to maximize the chances to detect or to identify expression changes in interesting genes. Affymetrix MAS5 algorithms were used to identify present transcripts, while RMA, GCRMA, VSN, and dChip methods were employed together with MAS5 to identify gene expression changes (see [28] for comparison among methods). Gene expression measurements were filtered to eliminate invariant probesets using the IQR>0.5 filter. MAS5 data was left unfiltered because application of the filter retained less than 10% of the probesets. The number of probesets that passed the filter for each processing methodology is detailed in Table 1. Filtered measurements were then employed to compare the gene expression in the velvet with the expression in the rest of tissues using paired t-tests. False discovery rate (FDR) method was applied to adjust the obtained p values for multiple testing (see [29] for discussion). However, the resulting number of genes with significant changes after adjustment was very low (see Table 1). In order to increase the number of possible candidates and, thus, to minimize the number of false negatives, we opted to use the list of genes with unadjusted significant differences for later analyses. Genes showing significant expression changes between tissues as well as those expressed in the velvet according to the MAS5 algorithm are detailed in the Table S1. Gene Ontology was used to identify genes codifying for secreted, extracellular or membrane proteins related to regeneration or nerve system growth (see Table 1 for details). Selected genes were gathered in a list containing 208 genes that was further annotated manually using the bibliography from PubMed [30] and the OMIM database [31]. A final list was obtained comprising 90 genes that includes several neurotrophins (*NT3* and *BDNF*) or their receptors (*NGFR*, *NTRK2*, and *NTRK3*), together with other trophic factors like *BMPs*, *TGFβ*, *Pleiotrophin*, *Midkine*, *Glial cell derived neurotrophic factor (GDNF)*, *Ciliary neurotrophic factor (CNTF)* or even *IGF* receptors. Guiding molecules were also identified, including netrines, ephrins, and several members of the semaphorin family, as well as several membrane or extracellular matrix (ECM) components like *laminin*, *cadherin 4*, *NRCAM* or *L1 cell adhesion molecule (L1CAM)*, all well-known axonal growth promoters. The list also includes other less known proteins, at least in what concerns to axonal growth, like *Meteorin*. All these genes are detailed in Table S2, which also summarizes the expression changes between tissues estimated after applying the different algorithms. As shown in table S2, estimated gene expression changes may differ among the different probesets that are available in the array for each gene. Given the cross-species character of the analysis, we did not quantify the expression changes leaving it for subsequent PCR analyses.

**Table 1:** Microarray data processing summary.

Algorithm	IQR>0.5	Comparison		p<0.05	adj p<0.05	GO:CC	GO:BP	Bibliography
MAS5 exp	54675	Velvet vs	FRNT	3547	0	731	57	93
			MSCH	4412	0	868	64	
			PED	3895	0	708	58	
Dchip	52339	Velvet vs	FRNT	3739	0	763	54	
			MSCH	6924	76	1386	88	
			PED	4584	0	879	56	
GCRMA	53642	Velvet vs	FRNT	1136	0	252	86	
			MSCH	1563	2	342	24	
			PED	1174	0	251	18	
RMA	53499	Velvet vs	FRNT	3557	0	784	55	
			MSCH	4412	42	939	76	
			PED	3895	1	853	56	
VSN	54350	Velvet vs	FRNT	3515	46	797	67	
			MSCH	4212	199	905	67	
			PED	3754	75	789	61	
MAS5 P/M/A		5038				999	76	

Summary of microarray data processing detailing the number of genes passing the different filters and analyses carried out. The first column indicates the preprocessing method employed. The second column (IQR>0.5) indicates the number of probesets with interquartilic range over 0.5, except in the MAS5 analysis where all probesets were included. The third column indicates the comparisons that were carried out (FRNT corresponds to frontal skin, MSCH to mesenchyme, and PED, to pedicle skin). P<0.05 indicates the number of probesets with significant expression changes according to a paired t-test. Adj p<0.05 represents the number of significant comparisons alter False Discovery Rate correction for multiple tests. GO:CC y GO:BP correspond to the number of genes with significant gene expression changes (without correction) that were included in any of the Cellular Components (GO:CC) or Biological Processes (GO:BP) lists according to the Gene Ontology terms. The last column, bibliography, details the number of genes that fulfilled all previous conditions and were more likely involved in relevant processes according to published references and the OMIM database. Last file algorithms, MAS5 P/M/A, corresponds to the probesets classified by MAS5 algorithms as present or marginal in at least 2 or the 3 samples of velvet.

### Real time PCR analyses of antler axon growth promoters

Gene expression from 15 of the 90 axon growth promoting proteins identified in the microarray studies were analyzed using qPCR methods. The selection (detailed in Tables 2 and 3) covers from well known trophic factors like *BDNF*, *CNTF*, *GDNF*, *FGF2*, or *Midkine*, to morphogens like *BMP2*, *BMP4* and *TGFβ* as well as ECM and basal laminae proteins like *Laminin*, *L1CAM* or *NR-CAM*, membrane molecules like *Cadherin-4*, and the less known axon growth promoters *Meteorin*, *Galanin (GAL)* or *Glucose-6 Phosphate Isomerase*. We did not include genes whose expression profile had been previously described in the antler tissues, like *NGF*, *NT3* or *Pleiotrophin*. However, we included factors previously analysed by immunohistochemistry (FGF2 or Laminin), or even studied by RT-PCR but without differentiating between antler tissues (*BMP2* and *BMP4b*). TGFβ1 was included as a control for our PCR analysis and to complete its expression profile by comparing with pedicle and frontal skin expression. Primers for the genes under study were designed based on cervid sequences or on conserved regions of *Bos taurus* sequences when cervid sequences were not available. Information on primer sequence, characteristics, amplicon, or sequence of origin is detailed in Table 2. Expression was analyzed in all samples used for the microarray but in P04, P05 and F06 samples that did not preserve enough material. RNA from new individuals was added to increase the number of samples *per* tissue. As indicated in Figure 1, expression values were measured in 5 velvet and mesenchyme samples as well as in 2 pedicle and 3 frontal samples.

**Table 2:** Real-time PCR primers.

Gene Symbol		Primer sequence	Tm (°C)	Theoretical amplicon sequence	Species/Accession number	Compared species
BDNF	F	CCACCAGGTGAGAAGAGTGATG	62,1	CCACCAGGTGAGAAGAGTGATGACCATCCTTTTCCTTACTATGGTTATTTCATACTTCGGTTGCATGAAGG	Bos taurus (NM_001046607)	BT HS RN CL SS
	R	CCTTCATGCAACCGAAGTATGA	58,4			
BMP2	F	TTTTTCCATGTGGAGGCTCTTT	56,5	TTTTTCCATGTGGAGGCTCTTTCAATGGACGTGTCCCCGCGTGCTTCTTAGACAGTCTG	Dama sp. (AJ001817.1)	–
	R	CAGACTGTCTAAGAAGCA	51,4			
BMP4b	F	GCGGGTCGGGAGTTGTAAA	58,8	GCGGGTCGGGAGTTGTAAAACCTCCGCGACCTTGAGACCTGAAACATGTGATGCGCCTTTTCTCAGG	Dama dama (S79174.1)	–
	R	CCTGAGAAAAGCGCCATCAC	59,4			
CNTF	F	CCGCCGGGACCTCTGTA	60,0	GTCACATTGCTTATTTGGACCAGTATAGACAGAAACAAACCCAGCTCACTTGTTTCCTGGGACAGTTGAG	Sus scrofa (U57644.1)	SS HS RN
	R	TCAGGTCTGAACGAATCTTCCT	58,4			
CDH4	F	CATCTCCGTCATGGACATCAAC	62,7	CATCTCCGTCATGGACATCAACGAGGCCCCCTATTTCCCCTCCAACCACAAGCTGATCCGCC	Bos taurus (XM_604152.4)	BT HS PT EC MM
	R	GGCGGATCAGCTTGTGGTT	63,5			
FGF2	F	GGGTCCGCGAGAAGAGTGA	61,0	GGGTCCGCGAGAAGAGTGACCCTCACATCAAACTACAACTTCAAGCAGAAGAGAGAGGGG	C. capreolus (AF152587.1)	–
	R	CCCCTCTCTCTTCTGCTTGAAG	62,1			
GAL	F	GAACTCGAGCCTGAAGACGAA	59,8	GAACTCGAGCCTGAAGACGAAGCCCGGCCAGGAAGCTTTGACAGACCACTGGCGG	Bos taurus (BC126798.1)	BT SS OA HS RN MM
	R	CCGCCAGTGGTCTGTCAAA	58,8			
GDNF	F	TGGATTTTATTCAAGCT	43,1	TGGATTTTATTCAAGCTACCATTCGAAGACTGAAAAGGTCACCAGAGAAACAAATGGCCGTGCTTCC	Bos taurus (XM_615361.4)	BT HS RN MM EC
	R	GGAAGCACGGCCATTTGTT	56,7			
	F'	GCCAGAGGACTACCCTGATCAG	64,0	GCCAGAGGACTACCCTGATCAGTTTGATGATGTCATGGATTTTATTCAAGCTACCATTCGAAGACTGAAAAGGTCACC		
	R'	GGTGACCTTTTCAGTCTTCG	57,3			
GPI	F	GTCCCCGGGTCTGGTTTG	60,5	GTCCCCGGGTCTGGTTTGTCTCCAACATTGACGGGACTCACATTGCCAAAACGCTGGC	Bos taurus (BC103416.1)	BT SS CF EC HS MM
	R	GCCAGCGTTTTGGCAATG	56,0			
LAMB1	F	CACAGCGCCTGGCAGAA	57,6	CACAGCGCCTGGCAGAAAGCCATGGACTTTGACCGAGATGTCCTGAGTGCCCTGGCTGAGG	Bos taurus (XR_042638.1)	BT HS EC RN MM
	R	CCTCAGCCAGGGCACTCA	60,5			
L1CAM	F	TAGCAGCCAGCCATCACTCA	59,4	TAGCAGCCAGCCATCACTCAACGGAGACATCAAGCCCCTGGGCAGCGATGACAGCCTGGCGGACT	Bos taurus (XM_001250423)	BT HS RN MM
	R	AGTCCGCCAGGCTGTCATC	61,0			
Midkine	F	CCGGGTGCCCTGTAACTG	60,5	CCGGGTGCCCTGTAACTGGAAGAAGGAGTTTGGAGCCGACTGCAAGTACAAGTTTGAGACCTGG	Bos Taurus (NM_173935.2)	BT HS MM RN
	R	CCAGGTCTCAAACTTGTACTTGCA	61,0			
MTRN	F	GTGAACCTACGGCCCAACAC	61,4	GTGAACCTACGGCCCAACACCTTCTCGCCCTCCCGGAACCTGACTCTGTGCATCAAGCCC	Bos taurus (XM_614019.4)	BT HS RN MM EC CF
	R	GGGCTTGATGCACAGAGTCA	59,4			
NRCAM	F	GGACACCCGGGAAGACTACAT	61,8	GGACACCCGGGAAGACTACATCTGTTACGCCAGATTTAATCACACTCAAACCATACAGCAGAA	Bos taurus (XM_876270.2)	BT HS MM RN
	R	TTCTGCTGTATGGTTTGAGTGTGA	59,3			
TGF B1	F	TCCTTTGACGTCACTGGAGTTG	60,3	TCCTTTGACGTCACTGGAGTTGTGCGGCAGTGGCTGACCCACAGAGAGGAAATAGAGGGCTTTCGCC	Cervus elaphus (DQ642715.1)	–
	R	GGCGAAAGCCCTCTATTTCC	59,4			
B-ACT	F	CAGATCATGTTCGAGACCTTCAAC	61,0	CAGATCATGTTCGAGACCTTCAACACCCCCGCCATGTACGTGGCCATCCAGGCTGTGCTGTCCCTGTATGCCTCTGGCCGCAC	Cervus elaphus (U62112.1)	–
	R	GTGCGGCCAGAGGCATAC	60,5			
GAPDH	F	AAGGCCATCACCATCTTCCA	57,3	AAGGCCATCACCATCTTCCAGGAGCGAGATCCCGCCAACATCAAGTGGGGTGATGCTGGT	Cervus elaphus (AY650282.1)	–
	R	CCAGCATCACCCCACTTGA	58,8			

Primers employed for real time PCR. The table details the gene symbol, primer sequence (F corresponds to forward primer and R to reverse), melting temperature (Tm, in degrees celsius), the hypothetical amplicon sequence together with the species of origin and its genbank accession number. The last column (compared species) indicate the species used in the comparison to identify conserved regions within the target sequence. BT corresponds to *Bos taurus*, OA to *Ovis aries*, SS to *Sus scrofa*, EC to *Equs caballus*, HS to *Homo sapiens*, RN to *Rattus norvegicus*, MM to *Mus musculus*, CL to *Canis lupus* y CF to *Canis familiaris*.

**Table 3:** Real-time PCR data summary.

		Tissue			
		VLVT	MSC	PED	FR
**Gene**	**BDNF**	32.5	*34.6*	32.8	32.4
	*CTNF*	*36.0*	*35.4*	*35.4*	*35.2*
	**MTRN**	30.1	29.2	30.9	30.9
	**NRCAM**	32.1	*34.3*	33.1	32.6
	**L1CAM**	33.3	*35.9*	31.5	31.3
	**MDK**	26.3	27.9	28.4	30.4
	*GAL*	*35.6*	*36.6*	*34.1*	*35.1*
	**GPI**	26.6	25.8	28.7	27.8
	**LAMB1**	27.0	25.2	28.1	28.2
	*GDNF*	*34.7*	*indet*	*34.4*	*34.2*
	**BMP2**	31.7	27.3	32.3	30.8
	*BMP4b*	*34.8*	*35.0*	*35.3*	*35.3*
	*CDH4*	*indet*	*indet*	*indet*	*indet*
	**FGF2**	26.5	28.4	26.9	27.0
	**TGFb**	28.2	26.4	29.3	29.6
	**βACT**	23.5	22.9	25.5	25.5
	**GAPDH**	22.5	21.8	24.2	24.3

The table details the average cycle threshold (Ct) for each tissue and gene analyzed. Ct values in italics underline type corresponded to genes considered not expressed for a given group of samples. This category includes all genes with Ct values over 35 (upper limit of detection) and those with undeterminable Ct values. GDNF was considered unexpressed in all tissues because the PCR system flagged all its measurements in spite that the CT values were just below 35. VLVT corresponds to velvet, MSC to mesenchyme, PED to pedicle skin, and FR to frontal skin.

Real time PCR data showed detectable gene expression (cycle threshold -Ct- below 34/35 cycles) for *BDNF*, *BMP2*, *FGF2*, *GPI*, *L1CAM*, *Laminin B1* (*LAMB1)*, *Meteorin*, *Midkine*, *NR-CAM*, and *TGFβ* in most samples (see Table 3), although *BDNF*, *NR-CAM* and *L1-CAM* showed expression values close to the detection limits in the mesenchyme samples. On the contrary, Ct values corresponding to undetectable gene expression were obtained when analysing *BMP4b*, *Cadherin 4 CDH4*, *CNTF*, *Galanin*, and *GDNF*. *GDNF* was considered unexpressed in all samples although some showed average Ct values below 35, because in all cases real-time PCR system considered their data problematic. In agreement, a second analysis using alternative primers did not yield detectable values for *GDNF* (data not shown). Dissociation curves showed a prevalence of single products with no primer dimer formation in all reactions (data not shown). Only *L1-CAM* reactions yielded second PCR products, probably corresponding to primer dimers.

Following normalization with *β-Actin* and *Glyceraldehyde 3-phosphate dehydrogenase (GAPDH)* endogenous controls, gene expression for each tissue was determined relative to velvet expression in the same individual using the semiquantitative ΔΔCt method (Ct values for each gene and sample are available in Table S3). Comparison between velvet and mesenchymal expression indicates that most axon growth promoters analyzed here are markedly overexpressed (over two-fold) in the velvet respect to the mesenchyme (*BDNF*, *FGF2*, *L1-CAM*, *Midkine*, and *NR-CAM*, see Table 4). Only morphogen *BMP2* appeared overexpressed in mesenchyme, whereas *GPI*, *LAMB1*, *Meteorin* and *TGFβ* did not show relevant variations. Most changes were statistically significant (p<0.05, Table 4) according to a Student's t-test regardless of whether *β-Actin* or *GAPDH* were employed as endogenous controls to normalize the data.

**Table 4:** Gene expression changes among axon growth promoters.

	mesenchyme		pedicle skin		frontal skin	
	βactin	GAPDH	βactin	GAPDH	βactin	GAPDH
**BDNF**	**4.568**	**5.677**	−2.047	−1.827	**−4.673**	**−3.426**
**BMP2**	**−2.888**	**−2.324**	−3.147	−2.809	**−2.514**	−1.843
**FGF2**	***1.970***	**2.473**	−2.948	**−2.603**	−4.890	−3.656
**GPI**	1.033	***1.297***	1.237	1.400	***−1.570***	−1.174
*L1CAM*	**4.423**	**5.623**	−8.381	−7.711	**−24.744**	**−18.898**
*LAMB1*	**1.563**	**1.883**	−1.466	−1.355	1.103	**1.458**
**MDK**	**3.686**	**4.686**	1.043	***1.134***	**3.757**	**4.919**
**MTRN**	−1.877	***−1.558***	−2.152	−1.991	**−2.861**	**−2.165**
**NRCAM**	**4.075**	**5.209**	−1.661	***−1.550***	**−3.738**	−2.707
**TGFβ1**	−1.020	1.253	−1.566	−1.462	1.106	1.528

Gene expression changes in the different tissues identified by qPCR. For each gene and tissue, the table indicates the mean fold change with respect to expression in the velvet. Changes written in bold characters indicate statistically significant changes according to a Student's t test. Data for L1-CAM and LAMB1 (in italics type) are included but not considered for subsequent analyses or discussion since their expression could not be confirmed by sequencing. Number of comparisons for mesenchyme = 5, for frontal = 3, and for pedicle = 2.

A comparison between gene expression in velvet and skin samples from pedicle and frontal showed an opposite trend, with most genes, including *BDNF*, *BMP2*, *FGF2*, *L1-CAM*, *Meteorin* and *NR-CAM*, appearing downregulated in velvet. Only Midkine expression appeared significantly upregulated in velvet compared to frontal samples, although it remained unchanged compared to pedicle. *GPI*, *LAMB1*, and *TGFβ* expression remained unchanged in the three tissues. Student's t tests confirmed the observed trend and showed that differences were significant mainly when velvet and frontal skin expression were compared (see Table 4). On the contrary, differences in expression between pedicle skin and velvet, although following the same trend as observed in the previous comparison, were smaller and so was its statistical significance. In fact, while frontal skin showed significant overexpression of *BDNF*, *BMP2*, *L1-CAM*, *Meteorin* and *NR-CAM* and significant underexpression of *Midkine* respect to the velvet, significant gene expression differences between pedicle skin and velvet were restricted to *FGF2* and *NR-CAM*.

The identity of the PCR products analyzed was confirmed sequencing velvet cDNA. Since the products amplified during the real time PCR were too short for optimal sequencing (around 60 bases long), new reverse primers were designed to extend the amplified sequences over 200 bases long (see table 5). Sequencing confirmed the expression of 9 out of the 11 genes being analysed, namely *BDNF*, *BMP2*, *FGF2*, *GPI*, *Midkine*, *Meteorin*, *NR*-*CAM* and *TGFβ1*. *L1-CAM* and *LAMB1* could not be sequenced even after trying several alternative primers, and despite the fact that *LAMB1* showed high levels of gene expression according to real time PCR analysis. Amplified sequences closely corresponded (sequence identity over 90%) to assayed genes from other mammalian species according to Blast results (Table 5). Sequence information for all analyzed mRNAs has been deposited in the GenBank [32] under accession numbers HM004074 to HM004081.

**Table 5:** Sequencing of axon growth promoters.

Gene		Primer	Amplified sequence	Observations
**BDNF**	F	CCACCAGGTGAGAAGAGTGATG	CCNCCAGGTGAGAAGAGTGATGACCATCCTTTTCCTTACTATGTTTATTTCATACTTCGGTTGCATGAAGGCTGCCCCCATGAAAGAAGCCAACCTCCGAGCACAAGGCAGCTTGGCCTACCCAGGTGTGCGGACCCGTGGGACTCTGGAGAGCATGAATG	98%(Eval = 4E-74) identity to *Bos taurus* BDNF (BC109860.1); >90% identity respect other mammals BDNF
	R	**CATTCATGCTCTCCAGAGTCCC**		
**BMP2**	F	TTTTTCCATGTGGAGGCTCTTT	TTTTTCCNTGTGGAGGCTCTTTCAATGGACGTGTCCCCGCGTGCTTCTTAGACAGTCTGCGGTCTCCTAAAGGTCGACCATGGTGGCCGGGACCCGCTGTCTTCTAGCGTTGCTGCTTCCCCAGGTCCTCCTGGGCGGCGCGGCCGGCCTCATTCCCGAGCTGGGCCGGAGGAAGTTCGCGGCGTCTGCTGGCCGCTCCTCATCCCAGCCTTCGGAC	98%(Eval = 3E-104) identity to Bos taurus BMP2 (BC134682.1); >92% identity respect other mammals BMP2
	R	**GTCCGAAGGCTGGGATGAG**		
**FGF2**	F	GGGTCCGCGAGAAGAGTGA	GGGTCCGCGAGAAGAGTGACCCTCACATCAAACTACAACTTCAAGCAGAAGAGAGAGGGGTTGTGTCTATCAAAGGAGTGTGTGCGAACCGTTATCTTGCTATGAAAGAAGATGGAAGATTATTGGCTTCGAAATGTGTTACAGACGAGTGTTTCTTTTTTGAACGATTGGAGTCTAATAACTACAATACTTACCGGTCAAGGAAATACTCCAGTTGGTATGTGGCAGTC	98%(Eval = 2E-109) identity to *C. capreolus* FGF2 (AF152587.2); >92% identity respect other mammals FGF2
	R	**GACTGCCACATACCAACTGGAGTA**		
**GPI**	F	GTCCCCGGGTCTGGTTTG	GTCCCCGGGTCTGGTTTGTCTCCAACATTGATGGGACTCACATTGCCAAAACGCTGGCCACCCTGAACCCCGAGTCCTCTCTCTTTATCATTGCCTCCAAGACCTTCACCACCCAGGAGACCATCACGAACGCAGAGACGGCGAAGGAGTGGTTTCTGCTGTCGGCCAAGGACCCTTCTGCAGTCGCGAAACACTTTGTTGCCCTGTCCACCAACACTGCCAAANNNANGNNGTTTGGAATTGATCCTCAAAATATGTTCGAGNTNNNNNNNNNNTAGGAGGCCGCTACTCGCTGTGGTCAGCCAT	98%(Eval = 2E-92) identity to *Bos taurus* GPI (AB036426.1); >85% identity respect other mammals GPI.
	R	**GATGGCTGACCACAGCGAGTA**		
**MDK**	F	TCCCTTTCTTAGCTTTGGCCTT	TCCCTTTCTTAGCTTTGGCCTTGGCTTTGGTCTTGGGGCTGCAGGGCTTGGTCACCCGGATGGTCTCCTGGCACTGGGCATTGTACCGCGCCTTCTTCAGGGTCCCCTGGCGGGCTTTGGTGCCTGTGCCATCACACGCCCCCCAGGTCTCAAACTTGTACTTGCAGTCGGCTCCAAATTCCTTCTTCCAGTTACAGGGCACCCGG	91%(Eval = 6E-112) idéntica a la secuencia de Midkine de *Bos taurus* (BC103416); >90% de identidad respecto a Midkine de otros mamíferos
	R	**CCGGGTGCCCTGTAACTG**		
**MTRN**	F	GTGAACCTACGGCCCAACAC	GTGAACCTACGGCCCAACACCTTCTCGCCCTCCCGGCACCTGACTCTGTGCATCAAGCCCCTCAGGGGCTCCTCGGGAGCCAATATTTATTTGGAAAAGACTGGAGAACTGAAACTGCTGGTGCGGGACGGGGACCTCGGGCCCGGCCAGGCGCCGTGCTTCGGCTTCGAGCAGGGGGGCCTGTTCGTGGAGGCAACGCCACAGCAAGACATCAGCAGGAGGACCACGGGCTTGCAG	96%(Eval = 6E-105) identity to *Bos taurus* Meteorin (XM614019.4); >89% identity respect other mammals Meteorin
	R	**CTGCAAGCCCGTGGTCCT**		
**NRCAM**	F	GGACACCCGGGAAGACTACAT	GGACACCCGGGAAGACTACATCTGTTACGCCAGGTTTAATCACACTCAAACCATACAGCAGAAGCAACCAATTTCTGTTAAGGTGATTTCAGTGGATGAATTGAATGACACTATAGCTGCTAATTTGAGTGACACTGAGTTTTATGGTGCTAAATCACATAGACAGAGGCCACCAGCATTTTTAACTCCAGACGGCAATACAAGT	99%(Eval = 5E-99) identity to *Bos taurus* NRCAM (XM_876270.2)); >93% identity respect other mammals NRCAM
	R	**GCCACTTGTATTGCCGTCTGGAG**		
**TBFB1**	F	TCCTTTGACGTCACTGGAGTTG	TCCTTTGACGTCACTGGAGTTGTGCGGCAGTGGCTGACCCACAGAGAGGAAATAGAGGGCTCTCGCCTCAGTGCCCACTGTTCCTGTGACAGTAAAGATAACACGCTTCAAGTGGACATTAACGGGTTCAGTTCCGGCCGCCGGGGTGACCTCGCCACCATTCACGGCATGAACCGGCCCTTCCTGCTCCTCATGGCCACCCCTCTGGAGAGACCCAGCACCTGCACA	99%(Eval = 2E-92) identity to previous *Cervus elaphus* TGFB1 sequence (DO_642715.1); >90% identity respect other mammals TGFB1
	R	**GTGCAGGTGCTGGGTCTCT**		

Sequencing of the genes analyzed by qPCR. For each target gene, the table details the employed primers, signalling in bold type the one included to increase sequence length for sequencing. The table also indicates the obtained product sequence and its identity with the most similar mRNA included in the GeneBank databases (% of identity and E value according to NCBI's BLASTn).

## Discussion

Every year of their lives, males from all deer species cast and regrow their antlers. During this regeneration cycle, antlers are innervated by sensory nerves growing at astonishing rates through deep vascular layers of the velvet. Paracrine regulation from the local antler environment acting on the growing axons may be responsible, among others, for the observed growth rate. In the present study, we analysed the gene expression of the antler tissues during the period of maximum growth trying to identify paracrine factors potentially involved in rapid nerve growth. Cross-species microarray analyses were performed to explore overall gene expression of different antler tip and related tissues. The data obtained allowed us to list 90 genes, potentially expressed in the velvet, that coded for extracellular and membrane proteins with known neurite promoting activity. Then, we used real time PCRs and sequencing techniques to analyse in detail the expression of 15 selected factors. These analyses yielded positive results for 8 of these genes, coding for BDNF, Midkine, basic Fibroblast Growth Factor (bFGF, also known as FGF2), BMP2 and TGFβ, Meteorin, and Glucose 6-Phosphate Isomerase, as well as the cell adhesion molecule NR-CAM. BDNF belongs to the well known neurotrophin family, key factors in the neurite outgrowth regulation of both embryonary and adult neurons [33], [34], [35] and pivotal in the nerve fiber regeneration of the peripheral nerve system [36], [37]. Midkine is a heparin binding protein with neurite outgrowth promoting capabilities for a wide variety of neuronal types [38], [39], including sensory neurons from DRGs [40]. FGFb is a member of the fibroblast growth factors family, with known neuronal survival and axonal growth promoting properties [41] as well as peripheral nerve regeneration promoter [42]. FGFb also enhances N-CAM, Cadherin and L1-CAM axonal growth promoting activity [43], [44], [45]. Bone Morphogenetic Protein 2 (BMP2) and TGFβ are morphogens from the transforming growth factor (TGF) family. The first one has an important role in axonal guidance [46] and neurite growth promotion [47], [48], [49]. On the other hand, TGFβ favors cell survival of different populations of neurons [50], [51], [52], promotes neurite outgrowth [53], and is overexpressed after peripheral nerve injuries [54], acting on neurons and, particularly, on Schwann cells [55]. Meteorin is a newly discovered protein that induces neurite outgrowth of dorsal root ganglion neurons *in vitro* [56], [57]. GPI (also known as neuroleukin) is an isomerase that within the cell catalyses the conversion of glucose-6-phosphate to fructose-6-phosphate, [58], [59], while outside the cell functions as a neurotrophic factor for spinal and sensory neurons, promoting the survival in culture of sensory neurons that are insensitive to nerve growth factor [60]. Finally, NR-CAM is a cell-adhesion molecule with a well defined role in axon guidance [61], [62], and also in promoting neurite growth of sensory neurons [61].

The expression in the antler of some of these factors has been previously reported. Basic FGF was studied by immunohistochemical techniques establishing its presence in the epidermis, the epidermal appendages and the deep vascular layers of the velvet as well as in the mesenchyme and its derived osteocartilage tissues [63]. Such a general distribution broadly agrees with our observations, which show the expression of FGF2 in both velvet and mesenchyme. TGFβ1 expression has been also previously analyzed in the antler tip by RT-PCR [18], observing similar levels of expression in the velvet and mesenchyme that fully agree with the present results. Finally, BMP2 expression in the antler was also established by Feng and colleagues [20] although they did not provide details on its tissue or cellular distribution. Our results confirm the antler expression of this morphogen and detail its expression pattern in velvet as well as in mesenchymal tissues.

Real time PCR analysis also indicated the expression of *GDNF*, *L1-CAM* or *LAMB1*, but we could not sequence their PCR products, lacking the necessary identity confirmation. This inconsistency was particularly striking in the case of laminin. Real-time PCR indicated high levels of *LAMB1* mRNA in the antler, in agreement with its previous detection using immunohistochemical techniques [23]. However, we were unsuccessful in sequencing it despite making several attempts with different sets of primers. Something similar occurred with cell adhesion molecule L1-CAM and trophic factor GDNF. Sequencing failure may be due to suboptimal primers, but up to 5 different primers were assayed to confirm *GDNF* without any success. It seems more likely that the expression levels of both factors were too low. In fact, real time analyses yielded very low signals for both *L1-CAM* and *GDNF*, close to the detection limits of the system.

Negative results were also obtained for *CDH4*, *Galanin*, *BMP4* and *CNTF*. In all four cases, expression was inferred from the microarray data but could not be confirmed by real-time PCR. Such disagreement is probably due to gene sequence differences between deers and the species used to design the microarray probes or the PCR primers. These differences would result in primers or probes not specific enough, leading to erroneous results. However, in the case of *CNTF*, contradiction between microarray and RT-PCR data arose most likely from the design of the probes used in the microarray. *CNTF* probeset from GeneChip U133 plus 2.0 array also interrogates on the expression of *Zinc finger protein 91*, which is co-transcribed with *CNTF* [64]. Thus, it could be that the microarray positive results refer to this zinc finger protein and not to *CNTF*, explaining why we did not observed any *CNTF* expression in the later PCR analyses. The case of *BMP4* is particularly interesting because although we could not detect it in our PCR analyses, this morphogen has been previously detected and sequenced in the antler [19]. Lack of positive results in our analyses cannot be caused by non-specific primers, since they were designed according to a deer sequence published by Feng and colleagues [19]. It is more likely that we failed to detect BMP4 expression because it is restricted to cartilage or other tissues included in the Feng and colleagues study but not in the present one.

In addition to the factors studied here, other molecules with axon growth promoting activity have been identified in the antler. A list of all these promoters is provided in Table 6 and comprises several neurotrophins and growth factors, such as FGF, EGF, Pleiotrophin, or PEDF, morphogens from the TGFβ family, members of the IGF family, together with Retinoic acid, and several substrate molecules like laminin and heparan sulphate. All these molecules have demonstrated axon growth promoting properties for different neuron types either in culture or *in vivo*, in most cases including sensory neurons like those innervating the antler. Lack of effect on growth of sensory axons has been determined for EGF [51] and BMPs [65], and is likely for PEDF, considering the high number of studies conducted on this molecule that showed no evidence of neurite outgrowth activity for sensory neurons. However, since EGF and PEDF are known mitogens for Schwann cells, they can indirectly promote axon growth in the antler acting on these glial cells [66], [67], [68]. Most of them are also known to promote axon regeneration following nerve system damage (see for example, [36], [37], [42], [69], [70], [71], [72], [73] with the exception of NR-CAM and Meteorin –this last molecule probably due to its recent discovery and the reduced number of studies. Moreover, several growth factors included in table 6 have been identified also in different models of epimorphic regeneration, like in the newt limbs, or in the fish fins. Classic among them are the retinoic acid [74], [75], the BMPs [76], [77] or the basic FGF [75], [76], [78], [79], but collagen [76], [80], heparan sulphates [81], laminin [82], and IGF [83] also participate in these regeneration processes. Even TGFβ has been proposed to contribute to processes of regeneration in echinoderms [84]. In most cases, these molecules are necessary for the organ regeneration to be completed or even initiated, but, little is known on their roles in the axon growth during the epimorphic regeneration process.

**Table 6:** Axon growth promoters identified in the regenerating antlers.

Molecules	Reference
Trophic factors	
Neurotrophins	
NGF	Li et al., 2007
NT3	Garcia et al., 1997
BDNF	Present study
Epitelial Growth Factors	
EGF	Barling et al., 2005
Fibroblast Growth Factors	
FGF2	Lai et al., 2007; present study
Insulin Growth Factors	
IGF-1	Francis and Suttie, 1998, Gu et al., 2007
IGF-2	Francis and Suttie, 1998
Transforming growth factor family	
BMP2	Feng et al., 1997; present study
BMP3B	Kapanen et al., 2002
BMP4	Feng et al., 1995
TGFβ	Francis and Suttie, 1998; Faucheux et al., 2004; Present study
Vascular Endotelial Growth Factors	
VEGF	Lai et al., 2007; Clark et al., 2006
Neurite Growth-promoting Factors	
Pleiotrophin	Clark et al., 2006
Midkine	Present study
Serpins	
Pigment Epiteliun growth Factor	Lord et al., 2007
Other trophic factors	
Glucose Phosphate Isomerase (GPI)	Present study
Meteorin	Present study
Retinoic Acid	Allen et al., 2002
Extracellular matrix	
Glycoproteins	
Laminin	Korpos et al., 2005; Present study*
Collagen type I	Price et al., 1996; Park et al., 2004
Glycosaminoglycans	
Heparan sulfate	Ha et al., 2005
Cell-Adhesion Molecules	
Immunoglobulins	
NR-CAM	Present study

The table includes the axon growth promoters known to be present in the regenerating antler and the corresponding reference.

Additional information on the activity of the different factors present in the antler may come from their spatial location and their relationship with the antler innervation. In this sense, most factors are present or expressed in the velvet, where nerves are located (see references in Table 6). Exceptions only include BMPs, laminin, and heparan sulphate for which no information on their antler distribution is available. Immunohistochemical and *in situ* hybridization studies indicate that many growth promoters from Table 6 present a high expression or immunostaining at the arterial smooth muscles from the inner vascular layer of the velvet dermis where most nerve fibres are observed. This is the case of NGF [8], and pleiotrophin [16] analysed by *in situ* hybridization, and EGF [85], basic FGF [63], and VEGF [63] (although not confirmed by Clark and colleagues using *in situ* hybridizations [16]), studied by immunohistochemistry. Such a spatial co-localization indicates a direct exposure of the axons to these molecules and would suggest a relevant role for them in the growth of the antler nerves, either promoting axon regrowth or coping with the axon trophic requirements.

It is evident that antler axons are exposed to several effective growth promoters during regeneration. But, are these promoters responsible for the very high growth rate observed in the antler nerves? Are regenerating antler axons exposed to growth promoters different from those present in normal or wounded skin? Skin expresses a number of trophic factors that help nerve regeneration during healing of cutaneous injuries. The abundance of axon growth promoters in the skin is such that it has been considered a neurotrophic organ [86]. In fact, all axon growth promoters included in table 6 are expressed in normal skin according to reports on individual molecules or to human gene expression profiles stored in the *GeneNote* database [87]. Moreover, our comparison of the gene expression between the antler velvet and the skin overlaying the antler pedicle or the frontal bone confirms this similarity. Data indicates that all but one growth promoters appear significantly repressed in the velvet with respect to the frontal and pedicle skin. Thus, although they probably contribute to nerve regeneration, they do not seem to play a key role in the rapid growth of the antler nerves. The only exception is Midkine, which was significantly overexpressed in the velvet respect to frontal skin samples, though not respect to pedicle skin. Somehow, Midkine expression profile fits with what can be expected from a molecule involved in the process of rapid axonal growth in the antler. However, the high expression levels of Midkine observed in the pedicle skin deserves some comment. Peculiar properties can be expected for the pedicle, the reservoir for the cells and tissues that builds up the antler every year. It could be possible that molecules like Midkine have to be present in the pedicle to activate or favor the growth of the different components of the antler, including its innervation. However, the observed expression levels are difficult to interpret without further analyses.

The results of the present study together with previous data show that, during antler regeneration, axons navigate in a local environment rich in growth factors and substrate molecules capable of promoting their rapid growth. Most of these molecules are also expressed by normal skin, where axons are able to regrow but do not attain the rates observed in the antlers. According to present data, among the factors identified in the antler, only Midkine is significantly overexpressed in the antler velvet with respect to normal skin, suggesting a possible role promoting fast axonal growth. However, antler regeneration is obviously a very complex process that we are far from complete understanding. We have just begun to analyze the axon regeneration in the deer antler, and many studies are still needed to identify other paracrine regulators as well as to evaluate the effect of factors like mechanical stretch, endocrine regulation, immune environment, or electric fields, which may also contribute or even determine the growing characteristics of the antler innervation.

## Methods

### Tissue sampling

Deer tissue samples came from biopsies of adult (4 or 5 years old) male individuals, kept at the Experimental Farm of the University of Castilla-La Mancha (Albacete, Spain). Samples corresponded to the tissues responsible for antler growth (mesenchyme and velvet) and the soft tissues overlying the bone (epidermis, dermis, and periostium) at the antler base or pedicle and over the frontal bone of the skull. All samples were harvested during the period of maximal antler growth (60 days after casting the previous antlers). To obtain the samples, the individuals were kept in a hydraulic restrainer and anesthetized with a low-dose combination of Xylazine (0.5 mg/kg of body weight; Calier, Barcelona, Spain) and Ketamine (1 mg/kg BW; Imalgene 100, Menial, Lyon, France). After taking the samples, anesthesia was reversed with Yohimbine (0.25 mg/kg BW; Sigma-Aldrich, St. Louis, MO, USA). Samples from the antler tip (mesenchyme and velvet) were dissected following the protocol described by Li *et al.* [10] while pedicle and frontal skin samples were taken using 4 mm. diameter biopsy punches (Stiefel, Madrid, Spain). All procedures were carried out by veterinaries and approved by the ethic committees of the Spanish Science Research Council, the Ministries of Environment and Agriculture, Fishery and Food, and Hospital Nacional de Parapléjicos, which approved this study (Ref ICS06025). Samples were frozen in liquid nitrogen and kept at –80°C until processing.

### RNA extraction and quality evaluation

Frozen tissues were crushed in a liquid nitrogen cooled mortar. Total RNA was extracted using TRIzol reagent (Invitrogen Life Technologies, Carlsbad, CA, USA) and purified using the RNeasy kit (Qiagen, Valencia, CA, USA). In all samples to be used in microarray analyses, RNA quality was assessed by electrophoresis in 2% agarose gels (Invitrogen Life Technologies, Carlsbad, CA, USA), containing 0.5 μg/ml ethidium bromide (Sigma-Aldrich, St. Louis, MO, USA). RNA quality was also evaluated by microcapilarity electrophoresis using the RNA 6000 total RNA Nano LabChip kit with the 2100 Bioanalyzer (Agilent Technologies, Santa Clara, CA, USA). This system provides estimations for the RNA concentration, the rRNA 18S/28S index, the value for the RNA integrity number (RIN) according to the algorithm proposed by Schroeder [88], and an electropherogram for each sample. These last graphical representations were also used to estimate the value of each sample's RNA on the RNA quality scale proposed by Copois and colleagues [89]. In the RNA samples used for real-time PCR, only RNA concentration as well as 260/280 and 260/230 absorbance indexes were calculated using Nanodrop ND-1000 spectrophotometer.

### Microarray hybridization

Deer RNA was hybridized to Affymetrix GeneChip U133plus 2.0 microarrays containing probes for more than 47000 human transcripts. RNA preparation, hybridization, staining, and scanning of the GeneChip® U133 Plus 2.0 were performed by Progenika Biopharma laboratories (Derio, Spain) following Affymetrix protocols. Internal controls were spiked in to control for proper hybridization, washing and scanning. Microarrays were scanned and analyzed using Affymetrix GCOS 1.4 software [90] to obtain the corresponding.CEL,DAT, and .EXP files for all samples. .CEL and .EXP files may be downloaded at the GEO database [27] under the accession number GSE20036.

### Microarray quality control

The presence of scratches or other artifacts on the arrays was assessed by visual inspection of the log transformed images (applying the R package *affy*, [91], [92] available at bioconductor website) as well as by the residual analyses developed by Reimer and Weinstein [93] (*affytools* available at [94]) and Bolstad and colleagues [95] (*affyPLM* package of R available at [96]). Image analysis was complemented with different methods using probe-level hybridization data, including:

- Comparison of the noise or raw Q, background, percentage of present calls, and scaling factor values for the different samples. All values obtained from the RPT files generated after GCOS analysis;
- Comparison of the GAPDH and β-actin 3′/5′ ratio and the expression values of the spikes bioB, bioC, bioD, and Cre. Values were calculated after processing microarray probe data using MAS5 [97], dCHIP [98] and RMA [99] algorithms implemented in the R package *affy;*
- RNA degradation plots and associated parameters [91]. Graphs and values were obtained using *Affy;*
- Residual analysis following the approach by Bolstad and colleagues [95] and implemented in the R package *affyPLM* [100].

### Microarray data analysis

Microarray analyses were carried out using a human platform to analyze deer samples. This approach, known as cross species analysis, assumes that the transcripts of one species will effectively hybridize to the probes of the array if both species share enough sequence similarity [25], [101]. Cross-species analyses may lead to problems of reliability and reproducibility in the obtained results. However, different studies have shown that the data so obtained are both valid [102], [103] and reproducible [104] although array sensibility decreases significantly. In this study we used human microarray U133plus 2.0, in spite of the release of an array for *Bos taurus*, more closely related to deers than humans. We did so because at the time we performed the analysis, coverage and annotation of the bovine array was very limited.

Preprocessing of the hybridization data was performed using 5 different, widely validated methods in order to maximize the chances of identifying expression changes. The methods employed were MAS5 (Microarray Suite 5, [97]), dChip [98], RMA and GCRMA [99] and VSN [105]. A detailed description of these methodologies can be obtained from Parmigiani and colleagues [106], from Gentleman and colleagues [107] or from the Bioconductor website [96]. Preprocessing of the data was carried out by analyzing the raw CEL files with the R packages, affy, vsn, and gcrma from Bioconductor. The data obtained included the expression values of all transcripts and an estimation of their presence or absence according to the MAS5 algorithms [97]. All posterior analyses were carried out in parallel with the gene expression values obtained after the different preprocessings.

Gene expression values were filtered using the R package Genefilter to eliminate invariant genes. We eliminated those genes whose gene expression did not show an interquatilic range higher than 0.5. dChip preprocessed data were log transformed before applying the filter. MAS5 data was not filtered because the number of transcripts that passed the filter was too low (3982) leading to a loss of potential relevant information. Filtered data (as well MAS5 unfiltered data) were used in the following differential expression analyses. Gene expression in the velvet was compared to all the other tissues using a paired analysis together with bayesian inference of variance following the methods implemented in the Limma package [108] of Bioconductor. Analyses allowed estimating the fold change and its significance according to the Student's t test as well as the False Discovery Rate developed for multiple hypothesis testing [29]. Genes showing significant expresion differences between velvet and other tissues according to the Student's t test or the FDR were annotated using Affymetrix Netaffx database [109] to identify the secreted, extracellular or membrane proteins according to the corresponding Cellular Component categories of the Gene Ontology (GO categories: Cell surface, Plasma membrane, Extracellular matrix, Extracellular region, Envelope) as well as related to regeneration, nerve growth or associated processes according to the Biological Process categories (GO categories: Nerve system development, Neurite morphogenesis, Axonogenesis, Axon extension, Regeneration, Tissue regeneration, Neurite regeneration, Axon regeneration). The same annotation scheme was followed with the genes identified as present (i.e. expressed genes) in the velvet according to the MAS5 algorithms. The resulting gene list was further annotated using Pubmed [30] and [31] searches in order to select the genes to be later analyzed by Real Time PCR.

### Real time quantification by-PCR

Relative quantification of gene expression was performed by real-time PCR (qPCR) analysis using SYBR green chemistry. Samples corresponded to those compared using microarrays plus some extra samples included for increasing the number of biological replications per tissue. Sequence data were lacking for most genes of interest. Thus we used Clustalx vs 3.0 [110] to compare mRNA sequences of different mammals (basically *Homo sapiens*, *Rattus norvegicus*, *Mus musculus* and *Bos taurus*) obtained from NCBI's Unigene and Entrez Gene resources in order to identify highly conserved regions. Primer3 web utility [111] was used to design the primers on these preserved sequences using *Bos taurus* sequence as a template. This program was also used to design the primers for the endogenous controls (GAPDH and β-actin) and other transcripts for which sequence information exists in any species of Cervidae. In all cases primers were designed to amplify an amplicon around 60 nucleotides long and a melting temperature close to 60°C. Primers were produced by BonsaiTech Company (Madrid, Spain). Information on the primer sequences, characteristics, amplicon, and sequence of origin is provided in Table 2.

Unamplified total RNA was used for realtime RT-PCR analyses following standard procedures. Briefly, complementary DNA (cDNA) was synthesized from 1 µg of total RNA pretreated with DNase (Roche) reverse transcribed using Moloney murine leukaemia virus (RT-MLV; Invitrogen) according to manufacturer's instructions. Each reaction was aliquotted and used as a template for qPCR. cDNAs were amplified using SYBR green following standard protocols (Applied Biosystems) with recommended buffer and dNTP concentrations (20 µl final reaction volume, 10 ng cDNA and 20 pmol/ml of primers and SYBR green master mix), using 7900HT Fast Real-Time PCR system (Applied Biosystems) with the implemented ΔΔCt routine (fast mode: 20 seconds at 95°C followed by 40 cycles of 1 sec at 95°C plus 20 seconds at 60°C). Serial dilutions of all cDNAs were previously amplified to establish the appropriate dilution to attain a detection range between 10 and 35 cycles for all endogenous and genes under study (1/50 dilution). Two endogenous controls (GAPDH and β-Actin) were amplified and measured in separate wells as real-time reporters. Primer specifity was tested by dissociation curves of the PCR products using the dissociation routine of the 7900HT system (reaction cooling to 60°C followed by slow heating to 95°C with continuous fluorescence measurement). Gene expression quantification was carried out in relation to the expression of the endogenous controls and the expression in the velvet following ΔΔCt method [112]. That is, for each gene and sample, a normalized expression was calculated as the difference between the cycle threshold (CT) value of the gene and the corresponding CT of the endogenous control. The obtained value, known as ΔCT is then used to calculate the fold change of the analyzed sample respect to a control sample using the formulae Fold change = 2^−ΔCTsample−ΔCTcontrol^: Fold changes were then compared among sample types using a Student's t test.

### PCR product sequencing

PCR products were sequenced to check their correspondence with the gene under study. Due to the small size (around 60 nucleotides) of the PCR products, new 3′ primers (shown in Table 5) were designed to increase the amplicon length up to 150–200 bases. Amplification was carried out using a PCR kit (Biotools) and a MyCycler Thermal Cycler (Biorad) employing the following cycle design: preheating during 3 minutes at 95°C followed by 30 cycles of 30 seconds at 95°, 30 s at 55°, and 45 s at 72°, to end with 10 minutes at 72° before lowering the temperature to 4°C. PCR products were electrophoresed in agarose gels, extracted and cleaned using QIAquick Gel Extraction Kit (Qiagen). The products so-obtained were subcloned in the prokariotic expression vector pGEM-Teasy (Promega), the plasmids amplified and purified in competent *E. coli* cells and the cDNA sequenced in a sequencing service (SECUGEN, CIB-CSIC).

## Supporting Information

Table S1 **Genes expressed in the antler velvet or with expression changes among sample types.** Microsoft excel file detailing all genes showing expression changes according to the different processing method or being present according to the MAS5 detection call. The first and the second column correspond to the Affymetrix probeset and the interrogated gene respectively. The following columns indicate which comparison and processing method (including MAS5 detection call for the velvet samples) detected significant changes for each gene/probeset. FRNT: frontal skin; MSC: mesenchyme; PED: pedicle skin.
(XLS)

Table S2 **Axon growth regulators identified by microarray analysis.** The table includes all genes coding for membrane or extracellular proteins involved in neurite growth or regeneration processes that were expressed in the antler velvet or showing expression changes between velvet and other antler tissues (mesenchyme, pedicle skin and frontal skin) according to the microarray data. For each gene, the table details its symbol, name, presence or absence according to MAS5 algorithms and its expression changes in the different tissues with respect to velvet (+ indicates overexpression in the velvet respect to the tissue being compared, - indicates repression, while +/- indicates contradictory expression change values depending on the probeset considered). The last column indicates the array probesets from which the data were obtained. Genes written in bold type were selected for expression analyses using real time PCR.
(XLS)

Table S3 **Real time PCR results.** Microsoft excel file detailing CT values for all samples and genes under study.
(XLS)
